# Jeans and language: kin networks and reproductive success are associated with the adoption of outgroup norms

**DOI:** 10.1098/rstb.2023.0031

**Published:** 2024-03-11

**Authors:** Qiao-Qiao He, Jie-Ru Yu, Song-Hua Tang, Ming-Yang Wang, Jia-Jia Wu, Yuan Chen, Yi Tao, Ting Ji, Ruth Mace

**Affiliations:** ^1^ College of Life Science, Shenyang Normal University, Shenyang, Liaoning 110034, People's Republic of China; ^2^ Key Laboratory of Animal Ecology and Conservation Biology, Institute of Zoology, Chinese Academy of Sciences, Beijing 100101, People's Republic of China; ^3^ College of Resources and Environmental Sciences, Gansu Agricultural University, Lanzhou 730070, People's Republic of China; ^4^ Life Science, Lanzhou University, Tianshui Road, Chengguan Qu, Lanzhou, Gansu Province 730000, People's Republic of China; ^5^ Department of Anthropology, University College London, London WC1H 0BW, UK; ^6^ IAST, Toulouse School of Economics, Toulouse, Occitanie, 31080, France

**Keywords:** globalization, culture evolution, frequency-dependent selection, kin network, reproductive success

## Abstract

Traditional norms of human societies in rural China may have changed owing to population expansion, rapid development of the tourism economy and globalization since the 1990s; people from different ethnic groups might adopt cultural traits from outside their group or lose their own culture at different rates. Human behavioural ecology can help to explain adoption of outgroup cultural values. We compared the adoption of four cultural values, specifically speaking outgroup languages/mother tongue and wearing jeans, in two co-residing ethnic groups, the Mosuo and Han. Both groups are learning outgroup traits, including each other's languages through contact in economic activities, education and kin networks, but only the Mosuo are starting to lose their own language. Males are more likely to adopt outgroup values than females in both groups. Females of the two groups are no different in speaking Mandarin and wearing jeans, whereas males do differ, with Mosuo males being keener to adopt them than Han males. The reason might be that Mosuo men experience more reproductive competition over mates, as Mosuo men have larger reproductive skew than others. Moreover, Mosuo men but not others gain fitness benefits from the adoption of Mandarin (they start reproducing earlier than non-speakers).

This article is part of the theme issue ‘Social norm change: drivers and consequences’.

## 1. Introduction

Globalization and colonization have led to the convergence of cultural practices [1] and endangered cultural diversity worldwide [2–9]. The loss of cultural diversity, particularly the loss of languages, has a long history and has been accelerating everywhere including in China, where Mandarin is becoming an increasingly predominant language. Certain traditional cultural values within small-scale groups may be more vulnerable than others to the effects of contact with other, more powerful groups [2,9]. Furthermore, cultural values, such as languages and dressing styles, that are more prevalent in a larger spatial domain may become more common as local groups expand their own spheres of interaction. Here we compare how and why different ethnic groups adopt their own cultural values, as well as those of other groups, at varying spatial scales, aiming to provide valuable insights into changes in the cultural norms of different scales in the process of globalization.

Frequency-dependent selection theory is an evolutional model that can be applied to explain why people in different groups adopt cultural values from outgroups or lose their own culture at different rates [4]. According to this model, individuals' social learning of cultural values is influenced by the frequency with which those values are adopted by others in the population. The ultimate reason is that the costs and benefits of adopting certain strategies are determined by their frequencies in the population, which might include facilitating communication, economic benefit, group-identity markers [10] and survival or reproductive success. That is, in certain contexts, it might pay for an individual to adopt the trait that is most common/rare in the population at that moment. The proximate mechanism is frequency-dependent transmission biases [11]. More common strategies have higher benefits in positive frequency-dependent selection, while in negative frequency-dependent selection, rare strategies have higher benefits. Favouring of rare traits may facilitate innovation; while bias in favour of common traits may facilitate conformity [12]. It should be noted that frequency-dependent transmission biases do not necessarily require benefits for the individuals adopting certain traits, as traits can spread in a population even if they are maladaptive at the level of individual fitness [13], at least in the short term. Although frequency-dependent transmission biases have received a lot of attention, relatively few studies have examined the fitness cost and benefits of language speaking or dressing styles of the individuals adopting them, such as in mate choice. If positive frequency dependence were a powerful and common process in cultural evolution then ultimately cultural diversity would be lost.

As globalization continues, connection to other groups increases, since people living in rural areas sample the frequency of cultural traits from a wider domain. Increased road density, trading, in-migration, travel, working in town, intermarriage between groups, exposure to social media and school attendance may all lead to greater exposure to outgroup cultural norms, potentially changing the frequency of exposure to the local groups' own/outgroup languages or dressing styles. Younger people are more likely to be involved in interactions with outsiders than elders and thus are more susceptible to these changes. Following more exposure to outgroup cultures, some individuals might therefore begin to adopt outgroup cultural values. Traits can spread through their social networks, such as the biological kin networks, affine networks or other networks.

Studies have found that ethnic groups with different kinship systems may have different cooperative network structures, especially in terms of who gets help from whom [14–16]. Population structure can shape the pool of cultural information and therefore constrain social learning, in the sense of changing connectedness [13,17]. Accordingly, groups with different kinship systems might also differ in transmission network structure. Kin-based and affine-based types of ties might be unequally important in social learning for females/males in some kinship systems.

Standard Mandarin Chinese (or Pŭtōnghuà), created based on the northern Han language in the first half of the twentieth century, has been spreading all over China as the official language and the main language of education, and has been adopted by more and more people living in both urban and rural areas. The Chinese government has put a lot of effort into spreading Mandarin in the whole country since 1956 [18]. Since 2000, to become a teacher, one needs to pass a national exam to obtain a certificate of Mandarin [19]. Since then, Mandarin has been used increasingly in school. In 2022, it was reported that the proportion of Chinese who can speak Mandarin has reached 80.72% [20], compared to about 73% in 2015 and 53% in 2000 [21].

Invented in America in the 1870s [22], denim jeans have become popular all over the world, including in China [23–26], often referred as to a type of ‘world dress' [23] or a ‘global phenomenon' [25]. In the early 1980s, jeans were adopted by China from Western countries [26], and have become increasingly popular in urban and rural areas ever since. The spread of jeans in China, as well as learning Mandarin, might bring some benefits to the individual adopting it, such as in mate choice.

The adoption of Mandarin and jeans might also cause the loss of local cultural norms. Southwest China has been predicted to be one of the areas with the loss of languages in the next 80 years [2], but different languages (or other cultural values) might be lost at different rates. Minority groups in China have been learning languages, dressing styles, and other cultural traits from the Han people, the largest ethnic group in China [27]. Intermarriage, trading and other kinds of interactions between groups might account for the adoption of majority Han cultural values in a minority group. Bargaining power has been shown to be important in explaining norm adoption during interethnic interaction [28]. Therefore, with lower bargaining power, minority groups may be more vulnerable to cultural loss during globalization and economic development than the Han. Nevertheless, interethnic interaction does not always lead to cultural loss [29,30]. Han people have also adopted some cultural values from minority groups, although to a relatively lower extent.

Here, we analysed how socio-demographic and network characteristics influence the adoption of four culture values of different spatial scales (table 1) in two co-residing ethnic groups with different kinship systems in a rural area of Sichuan Province, southwest China: the Mosuo and the Han. The local population has undergone rapid economic development and globalization from the 1980s. Mosuo and Han populations cohabiting in Lugu Lake provide a useful context for us to investigate how cultural norms changes after the frequency of cultural traits and relative bargaining power change during this process.

**Table 1.** Comparison of focal cultural characteristics of Mosuo and Han people.

		languages			**dressing style**	
		**Naru**	**Sichuan**	**Mandarin**	**jeans**	
group	Mosuo	own group	outgroup	outgroup	outgroup	
	Han	outgroup	own group	outgroup	outgroup	
spatial scale		town	province	nation	world	

The Mosuo speak the language ‘Naru', which is a Tibeto-Burman language of the Sino-Tibetan family [31]. The Han people in this province and two adjacent provinces speak Sichuan dialect (or Sichuan-Guizhou-Yunnan dialect). The linguistic difference between Naru and Mandarin is large, while that between Sichuan dialect and Mandarin is much smaller. Thus, Sichuan dialect speakers can understand and learn Mandarin much more easily than can Naru speakers. Although Han is the largest ethnic group in China (and in this province), locally the Han population is a minority, and Mosuo culture might therefore have had considerable influence on the local Han people. Mandarin has become more widespread here recently, as in other rural areas of China. When we did our first fieldwork here in 2007, few adults spoke Mandarin. However, this figure has changed after a decade, and all three languages have become important languages spoken by local Mosuo and Han people (see the Results for details).

Regarding dressing styles, the Mosuo have traditional clothing, which older women often wear on a daily basis. However others may now wear it only during festivals, weddings or tourism events. Local Han people no longer have a unique traditional dress, and their dressing styles are similar to Han in other rural areas of China. After 1949, Chinese men and women (of any ethnic group) often wore a simple blue suit of trousers and jacket, which we can still see occasionally in our study site today. The popularity of jeans in this region is a recent development, and its history is not entirely clear. During our first fieldwork in 2007 and 2008, we noticed that a few young men and women wore jeans. Local Han people and Mosuo men may be less reluctant to wear jeans than Mosuo women, as their original dressing styles included wearing trousers that are not so different from jeans.

We examined local Mosuo and Han people's adoption of the two outgroup cultural traits, speaking Mandarin and wearing jeans; and we also examined the adoption of two local cultural traits, speaking Sichuan dialect and speaking Naru (the Mosuo language) (table 1). Table 1 shows how these four cultural traits are seen at four levels of spatial scale, town, province, national and world. The Han follows patrilocal residence. Traditionally, the Mosuo have duolocal residence and visiting marriage (where spouses live apart), where marriage ties are relatively weak and paternal investment is limited, while investing in mating effort might lead to more reproductive partners [32,33]. Therefore, competition over mates might be stronger for Mosuo men than Han men. Mosuo men may learn to speak Mandarin, Sichuan dialect or wear jeans to gain some benefits in the marriage market. We predicted that Mosuo men are more likely to adopt outgroup cultural values and this may bring them some fitness benefits. Secondly, owing to different residential patterns, the importance of close biological kin and the affinal kin network in social learning to Mosuo and Han females or males might differ. We predict that affinal kin might be less important to the Mosuo than to the Han, as Mosuo generally live and interact more with their natal family. Finally, during economic development and globalization, the relative power of these two local populations might have experienced significant changes. It is possible that Han people are less likely to learn from the Mosuo than vice versa.

## 2. Methods

### (a) Study site

This study compared a matrilineal Mosuo (also called Moso or Na) population and a patrilineal Han population co-residing around the shores of Lugu Lake, Sichuan Province in southwest China. These are the two predominant groups in the area, which also contains several other minority ethnic groups. We studied all five Mosuo villages in this town. The total population is about 7000, of which the majority are Mosuo people. There are also a few Pumi, Yi and Tibetan people, etc. in these villages. According to historical accounts, the ancestors of matrilineal Mosuo arrived in this area around 500 AD. Although it is not clear when Han migrated into this area, these two populations seem to have lived next to each other for a long time. Studies in the 1950s recorded 51 Han households out of a total of 140 in one of our studied villages [34]. Some argue that the Han first immigrated into this area more than 170 years ago, and immigration has occurred multiple times throughout history, at different points in time [35].

The Mosuo and the Han have distinctly different kinship systems. In this population, about half of the married Mosuo adults have duolocal residence and ‘visiting marriage' (also called ‘zouhun' in Mandarin, or ‘sese' in Na), where men often do not live with their wife or children, but with their own matrilineal kin [31,33,36–38]. While in the Han, women usually disperse at marriage to their husband's household and live with or near their husband's kin (patrilocal or neolocal). However, intermarriages between Mosuo and Han are recorded from time to time [37].

This mountainous area was somewhat isolated historically, with horse trading as the main way to interact with cultural groups outside the area. Since the 1990s, transport links and the tourism economy have developed fast in this area, attracting lots of tourists from inside or outside of the province, as well as some in-migration for settlement. The recent immigration of outsiders led to an increase in intermarriage between Mosuo and Han, and a decrease in the traditional duolocal post-marital residence in matrilineal Mosuo society [37]. Nowadays, the younger generation receives formal schooling for at least 9 years, and children aged 3 or more go to kindergarten for 3 years. After graduating from school, a substantial proportion of young people work outside the town, moving to bigger cities either permanently or temporarily.

### (b) Data collection

Cultural characteristics of 1720 Mosuo and 712 Han people, who were present during the investigation, were collected in a single-round survey, along with a demographic and socio-economic survey of this population in 2017. The cultural survey was conducted immediately after the demographic survey, and included questions on languages spoken and dressing styles. Here we focused on four cultural characteristics varying across group and spatial scales (table 1), Mandarin (0 = cannot speak, 1 = can speak), Sichuan dialect (0 = cannot speak, 1 = can speak), Naru (0 = cannot speak, 1 = can speak) and wearing jeans (1 = never wear, 2 = rarely wear, 3 = often wear, 4 = always wear). We defined ‘1 = can speak' as being able to speak any of the focal language/dialect, even if only a few sentences.

We used demographic data to calculate reproductive success and to identify biological kin and affinal kin networks. We calculated the number of offspring living to age 15 for elder Mosuo and Han men and women born between 1911 and 1950. We computed the number of live children (of any age) and age at first birth for all the adult participants (aged 15 or more) of the cultural survey. We also defined the intermarriage status of all the participants (0 = no, 1 = intermarried with Han, 2 = intermarried with other ethnic groups).

### (c) Socio-demographic models

We conducted logistic regressions on the adoption of all four cultural values in Mosuo and Han females and males, separately. This allows us to examine sex and group differences associated with the social learning of each cultural trait. Nonetheless, the comparison of coefficients between models may be confounded by different residual variations across models (unobserved heterogeneity) [39]. Unobserved heterogeneity is well indicated by consistently higher/lower coefficients in one group than those in another group [39]. Thus, we compared the coefficients of the models of each subsample, and we found no sign of unobserved heterogeneity.

We examined several independent variables belonging to three levels, that is, individual, household and village level. The models included several variables: age (in years), occupation (0 = never had a job (never), 1 = had a full or part-time job before/now (ever)), education (in years, but with college and above combined), yearly household income (in six levels: 1 = ‘0–5000', 2 = ‘5000–10 000', 3 = ‘10 000–20 000', 4 = ‘20 000–50 000', 5 = ‘50 000–100 000', 6 = ‘greater than 100 000', Renminbi (RMB) yr^−1^) as socio-demographic predictors, controlling for villages (table 2).

**Table 2.** Sex and ethnic-group differences of four cultural characteristics. (Significant effects are in bold.)

group difference	male	female	sex difference (chi-sq)	
			$\chi_{1}^{2}$	*p*
Mandarin (yes)				
Mosuo	0.525 (387/737)	0.315 (309/982)	**77.378**	**<0**.**001**
Han	0.452 (146/323)	0.350 (133/380)	**7**.**591**	**0**.**006**
chi-sq $\chi_{1}^{2}(p)$	**4.799** (**0.028)**	1.561 (0.212)		
Sichuan (yes)				
Mosuo	0.943 (695/737)	0.849 (834/982)	**37**.**619**	**<0**.**001**
Han	0.991 (320/323)	0.974 (370/380)	2.789	0.095
chi-sq $\chi_{1}^{2}(p)$	**12.57** (**<0.001)**	**41.344 (<0.001)**		
Naru (yes)				
Mosuo	0.982 (724/737)	0.980 (962/982)	0.166	0.683
Han	0.319 (103/323)	0.192 (73/380)	**14**.**953**	**<0**.**001**
chi-sq $\chi_{1}^{2}(p)$	**576.453 (<0.001)**	**931.357 (<0.001)**		
jeans (often/always)				
Mosuo	0.666 (484/727)	0.523 (506/968)	**59**.**803**	**<0**.**001**
Han	0.533 (171/321)	0.458 (175/382)	**3**.**883**	**0**.**049**
chi-sq $\chi_{1}^{2}(p)$	**16.816** (**<0.001)**	0.403 (0.525)		
jeans (rarely/often/always)				
Mosuo	0.802 (583/727)	0.636 (616/968)	**54**.**979**	**<0**.**001**
Han	0.657 (211/321)	0.636 (243/382)	0.343	0.558
chi-sq $\chi_{1}^{2}(p)$	**25.358** (**<0.001)**	0.000 (0.993)		

Note that we combined two levels of occupation (electronic supplementary material, table S1) in the analyses, as the proportion of women who had a job ever/now are both low. Tourism involvement of households (0 = no, 1 = yes) was also controlled for as they are a measure of exposure to ‘outsiders' (table 2). We did not include the possession of a television in the models, since almost all households have televisions. Local people now use mobile phones widely, which we do not have data on. Nevertheless, occupation and yearly household income might be positively correlated to the possession of mobile phones. In the analyses of intermarriage status and cultural traits, only those who had ever married were included.

### (d) Network

We analysed ego-centric networks of both sexes of Mosuo and Han separately, and only included egos and alters with known cultural characteristics. In our network analysis of biological kin, we defined those closely related as relatedness more than or equal to 0.5, i.e. one's parents, full siblings and children; and defined affinal kin to current partners (‘relatedness' coded as 1), and current partners' parents and full siblings (‘relatedness' coded as their relatedness to one's partner). This resulted in two different networks, biological kin and affinal kin. The equal treatment of kinship enables the comparison of different effects on the spread of cultural traits in two kinship systems (Mosuo and Han). Owing to the limitations of random sampling, we are unable to accurately estimate the whole number of close kin adopting cultural focus traits. Therefore, we used node-label permutation tests [40] to analyse whether the observed assortment was significantly higher than chance. We conducted a permutation test on 1000 networks with the same pattern of connections as the observed networks, but with the cultural labels randomized across all nodes.

Moreover, to confirm the results of the permutation test, we also fitted two series of mixed-effects models for biological and affinal kinship networks, respectively, with the possession of each cultural trait as the dependent variable. The predictors were network variables, including alter's cultural traits and the number of kin/affines adopting focal traits. The models controlled for social-demographic factors (age, ethnicity and sex, education, occupation, household income and tourism involvement) and contained ego's identity as random effects. We also included the interaction of ethnicity with the number of kin/affines adopting focal traits. The biological and affinal kin models only included participants with one or more close biological/affinal kin surveyed (exclude: participants with no biological/affinal kin surveyed), respectively.

### (e) Reproductive success

We computed the multinomial reproductive success skew index (*M* value, [41,42]) for all the elder Mosuo and Han men and women, who had finished their reproduction (or at least most of the reproduction) before the governments' child policy in the 1980s, that limited births in this region. Based on Peter Nanac's binomial index, B [43,44] and Bayesian estimation, the *M* index accounts for variance in age across individuals and is not influenced by differences in sample or group size [41]. The government child policy started in the early 1980s, under which each Mosuo woman can only have up to three children and a Han woman can have up to two children. We also excluded people born before 1911 because of the small sample size.

We also conducted Poisson regressions on the relationship between each cultural value and the number of children alive (of any age), and used Cox regressions to analyse how cultural values are associated with age at first birth for all the adult participants. We also controlled age, age squared, occupation (0 = never, 1 = ever), education (in years), yearly household income (in six levels) and tourism involvement of household (0 = no, 1 = yes). The selection of variables was based on a minimal sufficient adjustment set selected from a simple directed acyclic graph that we illustrated (see the electronic supplementary material; the text for details of the method [45–48] and figure S7). To better examine how the local dynamics of these four cultural traits influence an individual's age at first birth, we only included people who started their reproductive careers after the first importation of Mandarin and jeans into China, i.e. people born after 1940 for the analyses of Mandarin, and those born after 1965 for the analyses of jeans. We also excluded people born outside the Lugu Lake area.

We combined the jeans-wearing variable into a binary variable (in two ways, either with 0 = low (rarely/never wear), 1 = high (often/always wear); or 0 = no (never wear), 1 = yes (rarely/often/always wear)). The pairwise deletion was applied to missing data. Demographic data enable us to identify any kinship relationship between all individuals. Pedigrees were created by linking each person in the census to their mother and father. Relatedness between each pair of individuals was calculated from the pedigree data using the ‘AGHmatrix' package [49]. Social network analyses were done with the ‘igraph' package [50]. No serious autocorrelation of predictors has been found by the ‘vif' function in the ‘car’ package [51]. Cox regressions were done with the ‘survival' package [52,53] and plotted with the ‘survminer' package [54]. Mixed-effect models were fitted with the ‘lme4' package [55]. Other figures are plotted with the ‘ggplot2' package [56], and the results of regressions were converted to tables by using the ‘textreg' package [57]. All data were analysed in R v. 4.2.2 [58].

## 3. Results

The electronic supplementary material, table S1 shows that male participants are better educated, and more likely to have a job other than being a farmer (now or ever), than are females in both Mosuo and Han, with sex differences being bigger in the Mosuo than in the Han. Mosuo males are slightly better educated than Han males in this area (less than 1 year difference). Although sex and group differences in yearly household income are statistically significant, the deviations are small.

### (a) Ethnic group differences and sex differences

A series of *χ*^2^-tests were carried out to compare ethnic group and sex differences. Relatively more males adopt outgroup cultural norms than females in both Mosuo and Han people, but sex differences are bigger in Mosuo than in Han (table 2). Almost all Mosuo males are capable of speaking the Sichuan dialect (94.3%, 695 out of 737), while 52.5% of them can speak Mandarin (387 out of 737), both significantly higher than Mosuo females (84.9% and 31.5%, respectively). Similarly, a higher proportion of Mosuo males wore jeans frequently (or ever) than did Mosuo females. Again, a significantly higher proportion of Han males can speak Mandarin and/or Naru than can Han females, although the sex differences are consistently slightly lower than in the Mosuo. The proportion of Han males who ever wear jeans is about the same as that of Han females.

Naturally, relatively more Mosuo can speak Naru and fewer can speak Sichuan dialect than Han (table 2). Moreover, there is no sex difference in speaking one's mother tongue for both Mosuo and Han people. Although females of Mosuo and Han are not different from each other in speaking Mandarin or wearing jeans, Mosuo males are always keener to adopt outgroup characteristics, such as Mandarin and jeans, than Han males (table 2). In contrast to our prediction, Mosuo women are not less likely to wear jeans than Han women.

The electronic supplementary material, text S1 and tables S2–S3 show that socio-demographic characteristics are associated with the adoption of outgroup cultural norms but are largely irrelevant to the ability to speak their own languages in both Mosuo and Han people. The only exception is that younger Mosuo females are less likely to be able to speak their own ethnic group language than elder Mosuo, and Mosuo males show the same tendency (although not significant). By contrast, young Han females and males do not differ from their elders in speaking their ethnic group language. Young people of both sexes of both ethnic groups are more likely to adopt all the outgroup norms, except young Han's speaking Naru (electronic supplementary material, figure S1, tables S2–S3). For people of both sexes in Mosuo and Han, schooling is positively correlated with speaking Mandarin but not to any of the other languages or to wearing jeans (electronic supplementary material, figure S2, tables S2–S3). Employment is correlated with the adoption of outgroup cultural norms too, but works differently for Mosuo and Han males or females (electronic supplementary material, figure S3, tables S2–S3, text S1). At the household level, household income and involvement in tourism also seem to matter for the adoption of outgroup cultural norms (electronic supplementary material, figures S4–S5, tables S2–SS3, text S1). There are also some differences from village to village.

Not only are Mosuo people learning the local Han people's language, but also the Han are learning the Mosuo language Naru, although with a much lower frequency (table 2; electronic supplementary material, figures S1–S5). The electronic supplementary material, table S4 shows that intermarriage between these two ethnic groups explains some of the variance in speaking Naru for both Han men and women, but has nothing to do with the Mosuo's adoption of the Sichuan dialect. Moreover, after controlling for intermarriage, village-level effects remain on whether Han people can speak Naru or whether a Mosuo female can speak the Sichuan dialect.

### (b) Networks

We also built biological- and affinal-kin networks among all of the cultural-survey participants (electronic supplementary material, figure S6, table S5), and tested whether culture norms spread on these kin networks. We analysed Mosuo females', Mosuo males', Han females' and Han males’ networks separately, where their connections can be of any sex and any ethnic group.

**Figure 1.**
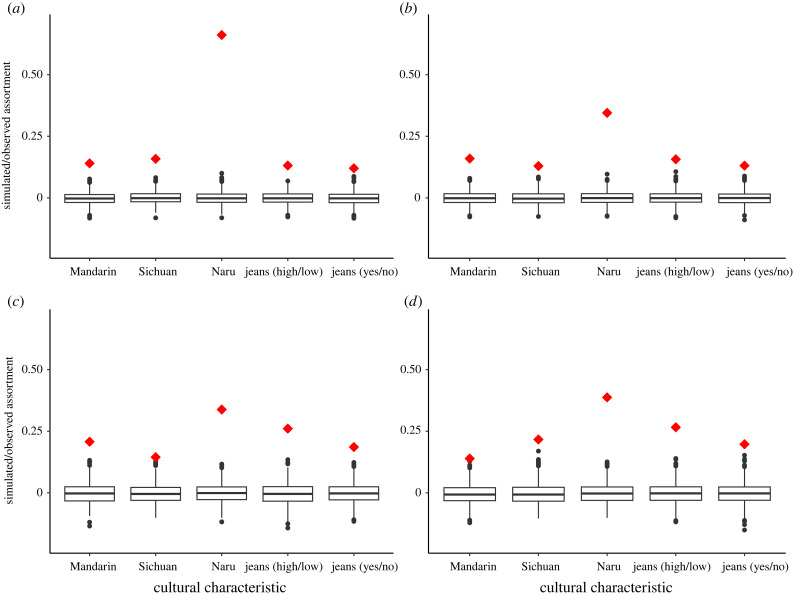
Assortativity on whether Mosuo (*a*) females and (*b*) males; and Han (*c*) females and (*d*) males possess focus cultural characteristics with their connections in close kin networks (*r* ≥ 0.5). For sample size, see the electronic supplementary material, table S6. Red diamonds represent the observed levels of assortment. Boxplots represent null distributions generated from a permutation test of 1000 networks with the same pattern of connections as the observed networks, but the cultural labels are randomized across all nodes. The box represents the interquartile range (IQR); the line through the middle is the median. Whiskers extend to 1.5 × IQR; outliers are plotted as dots.

**Figure 2.**
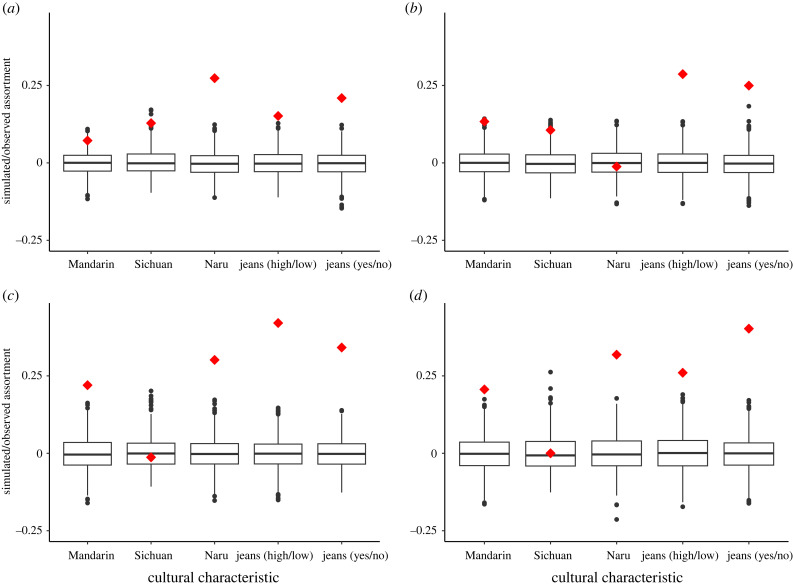
Assortativity on whether Mosuo (*a*) females and (*b*) males; and Han (*c*) females and (*d*) males possess focus cultural characteristics with their connections in affinal-kin networks (partners, partners' parents and full siblings). For sample size, see the electronic supplementary material, table S6. Red diamonds represent the observed levels of assortment. Boxplots represent null distributions generated from a permutation test of 1000 networks with the same pattern of connections as the observed networks, but the cultural labels are randomized across all nodes. The box represents the IQR; the line through the middle is the median. Whiskers extend to 1.5 × IQR; outliers are plotted as dots.

Some outgroup cultural characteristic of people in a network shows assortment higher than chance, indicating that this characteristic may spread on the network. Assortment of those four cultural characteristics varies from network to network, with Mosuo but not Han people showing some sex differences. Blood kin and affinal kin appear to both be very important in learning cultural norms, with blood kin being almost equally important for both ethnic groups and affinal kin more important for Han people. Figures 1 and 2 show the results of a permutation test run on each of these networks for each cultural characteristic. The probability of this level of assortment on all four characteristics occurring by chance is unlikely in blood-kin networks of both Mosuo and Han females and males, with the assortment of Mosuo females/men's speaking Naru much higher than other characteristics in it (figure 1*a–d*). Similarly, Mosuo and Han males/females' frequency of wearing jeans also shows a high level of assortment in their affinal-kin networks (figure 2*a–d*). Nonetheless, when it comes to language, outgroup languages (Mandarin and Naru) but not the mother tongue (Sichuan dialect) of Han people show high levels of assortment in their affinal-kin networks (figure 2*c,d*). Mosuo people show no assortment on any languages in affinal-kin networks, except that Mosuo females show a higher assortment on speaking Naru.

We also fitted two series of mixed-effect models with network variables (alter's cultural traits, the number of biological/affinal kin adopting the focal cultural trait and their interaction with ethnicity) as predictors, and adoption of focal cultural traits as dependent variables (electronic supplementary material, tables S7–S8). The models also controlled for social-demographic factors. Note that the number of biological/affinal kin adopting focal cultural traits is not exactly correct, because we only know the cultural traits of kin who participated in the survey. Therefore, the results might not be as reliable as those of permutation tests. Nevertheless, the results confirm that kinship networks are important for the adoption of outgroup cultural traits. Similar to the results of permutation tests, blood kin are similarly important to two ethnic groups and affinal kin are more important to Han people (indicated by negative interaction between Mosuo ethnic and the number of affinal kin adopting focal cultural traits; electronic supplementary material, table S8).

### (c) Reproductive skew and cultural differences in reproductive success

**Figure 3.**
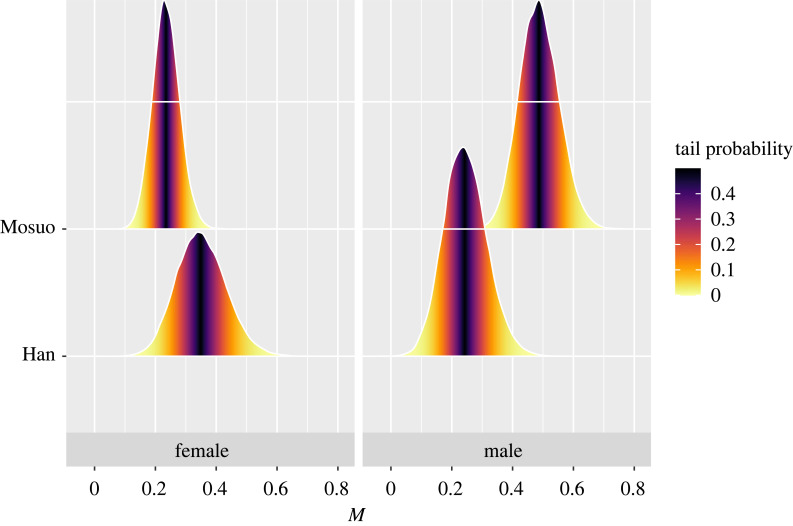
Posterior estimations of skew of reproductive success (measured by number of children living to 15 years) are larger in Mosuo men (*n* = 485) than Han men (*n* = 181; mean difference = 0.24, 90% confidence interval (CI) = (0.08, 0.40)), but are the same in Mosuo women (*n* = 576) and Han women (*n* = 169; mean difference = −0.12, 90% CI = (−0.26, 0.04)). We only analysed people born between 1911 and 1950.

As predicted, sexual selection as measured by reproductive skew appears to be the strongest in Mosuo men. We calculated group differences using posterior estimations. Figure 3 shows that elder Mosuo men have larger reproductive skew than Han men, but Mosuo women do not differ significantly from Han women in the levels of reproductive skew.

**Figure 4.**
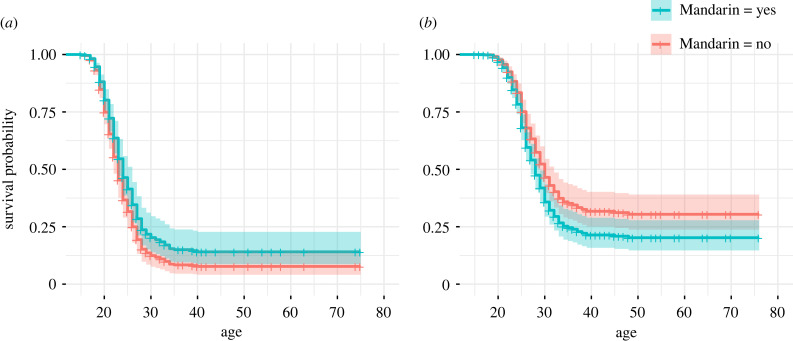
Cox regression plot of first birth varying with age for (*a*) Mosuo women and (*b*) Mosuo men born after 1940 in the Lugu Lake area. Mosuo male speakers start to reproduce earlier and Mosuo female speakers start to reproduce later than non-speakers of the same sex. The models controlled for age (in 2017), age squared, education, occupation status and yearly household income.

The electronic supplementary material, table S9 and figure 4 show that speaking Mandarin is associated with reproductive benefits to Mosuo men in earlier first reproduction, even after controlling for age, age squared, education, occupation status and household income. By contrast, managing Mandarin is not obviously beneficial to others in terms of reproduction; for Mosuo women, Mandarin speaking is associated with later first birth (possibly owing to an association with education, see below). Other cultural values are not correlated with the reproduce success of Mosuo men or women (age at first birth, electronic supplementary material, table S9; number of alive children (of any age), electronic supplementary material, table S11). Neither adoption of Mandarin nor any other cultural values is associated with age at first birth (electronic supplementary material, table S10) or number of living children (of any age, electronic supplementary material, table S12) of Han men and women. Higher levels of education are only associated with smaller family sizes and later first birth for women but not for men of both groups.

We also examined whether the benefits of speaking Mandarin on age at first birth in Mosuo men can be explained by the advantage of attracting mates from outside of the group (through facilitated communication). The prediction was not supported. We conducted a Cox regression on Mosuo men's age at first birth, with intermarriage status controlled. Contrary to the prediction, Mosuo men who married Han women (or those of other ethnic groups) start reproduction later than those married to Mosuo women (electronic supplementary material, table S13).

## 4. Discussion

The process of globalization often results in cultural convergence and a loss of cultural diversity, but group differences may exist and cultural values with different spatial scales might spread or be lost at different rates. Frequency-dependent selection can help explain these phenomena [4]. We compared the adoption of four cultural values with varied spatial scales among two co-residing populations, the Mosuo and Han in rural southwest China, which is considered a potential hotspot for the loss of cultural diversity [2]. We find surprising patterns in sex and group differences. Our findings support the frequency-dependent selection model, suggesting that factors such as age, sex, education, employment, involvement in tourism, intermarriage and connections in kin networks significantly influence the adoption of cultural values from outside the group. Although Mandarin and jeans have relatively different diffusion processes, the factors associated with their adoption are not very different.

### (a) Group and sex differences

Our results indicate that the cultural norms of the Mosuo, a minority population nationally with restricted geographical distribution, are more vulnerable to change than those of the Han population. There are significant differences in the adoption of our focal cultural values across different groups and genders. Both populations are influenced by cultural values from outside as well as those from each other. However, it appears that the Mosuo are more influenced by Han culture than the other way around.

Furthermore, Mosuo males are the most receptive to learning cultural values from outside sources, followed by Han males. Females in both groups are less likely to adopt outgroup cultural values than males, and there is no significant difference between females of both ethnic groups. Moreover, our findings suggest that young Mosuo individuals are less likely to be able to speak their native language compared to older generations, while the same trend is not observed among the local Han population.

Differences between Mosuo males and Han males cannot be explained by the similarity between their own languages and Mandarin, which should predict exactly the opposite since it should be easier for Han people to learn Mandarin than for Mosuo people. Sexual selection could help explain why differences in the adoption of Mandarin or jeans exist in males but not females of these two groups. We found that reproductive competition is stronger for Mosuo men than Han men or women of both groups, and the adoption of Mandarin brought Mosuo men's age of first birth earlier but not others', although it is not associated with the number of living offspring (perhaps not surprisingly given the birth policy and demographic transition). These results suggest that Mosuo men managing outgroup cultural values may have some advantage in attracting spouses and can start to reproduce earlier. This appears to be a general advantage, rather than just about attracting mates from outside of the group. Mosuo female speakers of Mandarin start to reproduce later, which might reflect that the influence of education and demographic transition is larger in women than men; the negative effects of education on reproductive success are only found in women.

Speaking Mandarin might act as an honest signal for Mosuo men, which indicates their ability to manage new knowledge. Surprisingly, wearing jeans does not affect one's reproductive success. The explanation might lie in the fact that learning a language is more costly and serves as a better signal of mate quality than wearing jeans. Another possibility is that our cross-sectional data may be less capable of fully capturing the relationship between reproductive success and rapid cultural diffusion processes, such as wearing jeans. For example, some of the current jeans' wearers might have started wearing them only recently, and their reproduction may be largely irrelevant to the wearing of jeans. Although cross-sectional data can provide some clues about individuals' willingness and capability to adopt outgroup cultural traits, the relationship between jeans and reproductive success might be better understood with longitudinal data. This limitation may not apply to Mandarin, because learning to speak Mandarin is a relatively slow and costly process.

Labour division might also have something to do with patterns of cultural trait adoption. Labour division is influenced by dispersal patterns and kinship systems [59]. Mosuo men used to do lots of horse trading in the past and were reported to be in charge of communication with external parties, while Mosuo women are more focused on the management of the household. As a result, Mosuo males might be more likely to be exposed to and be keener to learn from outsiders than Mosuo females. However, the labour division between Han males and females might be less than in the Mosuo, and thus sex differences can be smaller. However, this hypothesis cannot explain why differences in the adoption of outgroup cultural values are only found in males of these two groups but not in females.

### (b) Frequency-dependent selection

We found that schooling, employment, household wealth and exposure to tourists all increase the probability of adopting outgroup cultural values in both groups to some extent, supporting the frequency-dependent selection theory. To be specific, all four variables above changed the frequency of the trait sampled by the individual. More importantly, the interactions with outsiders and their cultural values impose some benefits for local people who adopt these values, especially in the sense of facilitating communication and integration and bringing access to new sources of income.

In addition to the improved bargaining power hypothesis [28], the demographic/subsistence model and ‘élite dominance' model in linguistics [60] are two hypotheses which might also help to explain social learning by looking at the frequency-dependent cost and benefits of adopting outgroup cultural values. The former argues that the emergence of a new language is a result of a large influx of people who speak the new language and often introduce a new mode of subsistence. On the other hand, the second model proposes that a small group of highly organized people, who speak a different language, arrive from outside the territory and can dominate the existing population owing to their military effectiveness, thereby bringing the population under their control. Both models, together with the bargaining power hypothesis, highlight a form of power of the new group over the existing group, which could stem from a new mode of subsistence and higher population growth rate, or better societal organization and military power. Local populations might then benefit from learning languages and even other cultural values such as the dressing styles of the more powerful group, in the sense of facilitated communication with the new group and gaining access to their resources. It is also possible that the frequency of cultural values changes following the arrival and population growth of the new group, so new cultural variants are acquired by a more neutral copying process. As the connection to Han people from outside increases, the local Han might have become a more ‘élite' (powerful) group. This can help explain why the Mosuo are more influenced by Han culture than the other way around, and why the Han are not losing their own language as much as the Mosuo. However, these models cannot explain why the Mosuo women do not differ from Han women in the frequency of speaking Mandarin and wearing jeans.

On the other hand, as a minority group in this area, some Han people marry Mosuo and learn the Mosuo language from their Mosuo affinal kin, their own biological kin, or during work. Intermarriage has been proven to facilitate the learning of languages, while knowing a second language also facilitates mixed marriage [61]. However, young Han men are less likely than old ones to know Naru after controlling intermarriage. This result might indicate that the Mosuo and their language have begun to lose their importance to other ethnic groups in this area, as we previously found in residential patterns [37].

### (c) Kinship and kin networks

Population structure has an important role in cultural transmission [17]. Affinal kin are predicted to be less important for the Mosuo than the Han, because marriage ties are weaker in the Mosuo, who are on average less likely to reside with or live near their affinal kin, but more like to reside with their biological kin than the Han. Permutation tests in two kin networks support this prediction. Assortment on four cultural values was consistently higher than chance in close-biological-kin networks for both sexes in the Mosuo and Han, and the highest assortment occurred in the Mosuo speaking their own language. Mixed-effect model analyses also confirmed this result. This result emphasizes the importance of family in the conservation of small-scale languages in circumstances where that language is not used in school. In some areas of China, regional languages have been taught alongside Mandarin [62], which should help conserve languages too; while in affinal-kin networks, as predicted, the assortment was more likely to be observed in Han than Mosuo (women's and men's) networks. Although, unlike in biological-kin networks, assortment in affinal-kin networks probably reflects assortative mating rather than just social contact. Furthermore, longitudinal data are needed to reveal the diffusion of cultural characteristics on kin networks.

In conclusion, our results show that a small-scale human population the Mosuo suffered the loss of cultural traits from globalization more than a larger ethnic group (the Han) with a wider geographical range, even though that group was a minority locally. Mosuo males appear to be more likely to adopt outgroup cultural values than Han males, while females of both groups do not differ from each other. This may be associated with differences in the strength and the benefit of sexual selection. Mosuo men experienced the strongest sexual selection and appear to be the only subgroup that benefits in terms of reproductive success from the adoption of outgroup cultural traits. Both individuals' socio-demographic characteristics and connections in kin networks influence the adoption of outgroup cultural values, supporting the frequency-dependent selection model. However, the current study is a cross-sectional study. Further works could analyse longitudinal data on the dynamics of cultural values and test how the changes in socio-demographic characteristics and kin networks influence culture-norm changes in globalization. Measures of an individual's linguistic competence on a larger scale can provide more reliable information than the binary response we used in this work.

## Data Availability

Data is available from the Dryad Digital Repository: https://doi.org/10.5061/dryad.v41ns1s27 [63]. Supplementary material is available online [64].
